# GeminiOne transcatheter edge-to-edge repair: comparative hemodynamic and biomechanical evaluation

**DOI:** 10.3389/fcvm.2025.1558454

**Published:** 2025-06-25

**Authors:** Kai Wang, Dongyang Xu, Bowen Xiao, Zhaoming He, Jianfong Tan, Saibal Kar

**Affiliations:** ^1^ Advance Technology Department, Peijia Medical Limited, Suzhou, China; ^2^ Department of Biomedical Engineering, Worcester Polytechnic Institute, Worcester, MA, United States; ^3^ Department of Mechanical Engineering, Texas Tech University, Lubbock, TX, United States; ^4^ Sierra Valve LLC, Irvine, CA, United States; ^5^ Department of Cardiology, Los Robles Regional Medical Center, Thousand Oaks, CA, United States

**Keywords:** mitral valve, TEER, MitraClip, GeminiOne, *ex vivo* simulator

## Abstract

**Background:**

Transcatheter edge-to-edge repair (TEER) is frequently used to treat mitral regurgitation (MR) patients. Despite its widely reported efficacy, complications such as single-leaflet device attachment (SLDA) and loss of leaflet insertion (LLI) can lead to recurrent MR, which compromises the clinical outcomes.

**Objectives:**

This study compares the acute MR reduction and leaflet anchoring stability of a novel TEER device, GeminiOne (GEM), and MitraClip (MC).

**Methods:**

In this study, *ex vivo* benchtop degenerative mitral regurgitation (DMR) and functional mitral regurgitation (FMR) models were used to evaluate the acute effectiveness of MR reduction by MitraClip XTW and GeminiOne 0626 in a BDC pulsatile flow duplicator. Furthermore, a benchtop study was performed to compare leaflet anchoring stability between XTW and GEM0626, in an attempt to investigate the likelihood of post-procedure leaflet detachment.

**Results:**

The results of the pulsatile flow evaluation from the DMR and FMR model demonstrate that both TEER devices effectively reduced the regurgitant fraction (DMR vs. GEM0626 vs. XTW, 59.21 ± 10.29% vs. 35.73 ± 6.62% vs. 43.50 ± 8.89%; FMR vs. GEM0626 vs. XTW, 56.99 ± 8.74% vs. 27.99 ± 11.30% vs. 28.13 ± 10.64%). However, in the leaflet stability study which compared the various TEER devices under full grasp and partial grasp conditions, the detachment force of the anchored leaflet for GeminiOne is significantly higher than that of MitraClip, especially for the partial grasp (full grasp detachment force: 7.89 ± 2.42 vs. 6.36 ± 0.96 N, *p* = 0.1214; partial grasp detachment force: 6.03 ± 2.05 vs. 2.97 ± 0.76 N, *p* = 0.0021).

**Conclusion:**

In the *ex vivo* pulsatile experiments, both GEM0626 and XTW are effective in terms of acute reduction of MR caused by DMR and FMR. However, in an anchored leaflet stability study, under partial grasp conditions, GEM0626 demonstrated a significantly higher leaflet detachment force. The better anchored stability of GeminiOne TEER may have long-term clinical benefits for MR treatment.

## 1 Introduction

**Figure 1:**
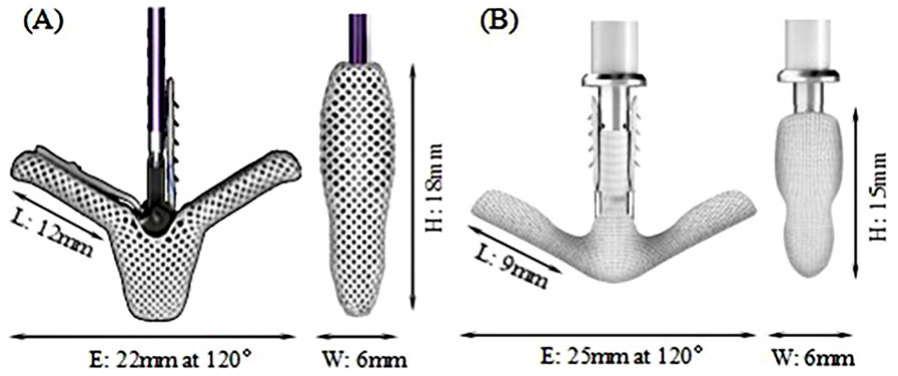
**(A)** MitraClip (MC) XTW. **(B)** GeminiOne (GEM) 0626.

Mitral regurgitation (MR) is a common valvular disease that can lead to heart failure, pulmonary edema, and life-threatening conditions (1–3). In recent years, transcatheter edge-to-edge repair (TEER) has been increasingly used in functional mitral regurgitation (FMR) and has been evaluated against optimal medical therapy resulting in an upgrade of the recommendation (4–7). The MitraClip (MC) system (Abbott, Santa Clara, CA, USA) is the first-of-its-kind device in the field of transcatheter mitral valve treatment. In the MC TEER procedure, native mitral leaflets are captured and secured between the TEER clip arms (closed) and grippers resulting in the reduction or elimination of MR. Although the safety and efficacy of the TEER treatment have been improving over the recent years with newer versions of TEER device and with better accumulated experiences by the physician (8, 9), single-leaflet device attachment (SLDA) and loss of leaflet insertion (LLI) remain as the main complications associated with TEER procedures (10–12). These complications may occur during the procedure and/or beyond hospitalization (10). Sugiura et al. (13) found that leaflet tear and LLI were the most common morphologies associated with recurrent MR in patients with degenerative mitral regurgitation (DMR). Residual leaflet prolapse or tear can progress over time, gradually leading to residual MR and recurrent MR. Ikenaga et al. (14) also studied the causes of MR recurrence in primary MR. They identified worsening leaflet prolapse, subsequent leaflet tear, and SLDA as the main factors contributing to MR recurrence. SLDA and LLI can happen due to either incomplete or inadequate grasping of the leaflets or, in the case of fully grasped leaflets, native leaflet tears or perforations (12–15). Therefore, the TEER device must provide a stable and secured anchoring of the native leaflets in its closed configuration to minimize MR to avoid subsequent residual MR to provide long-term sustainable clinical benefits. The MC system has undergone several upgrades to simplify the procedure and enhance the MR reduction while minimizing the occurrence of complications, with G4 being the latest generation. XTW (long wide model), shown in Figure 1A, has a nominal arm length is 12 mm and a width of 6 mm and consists of six pairs of frictional elements (16) on each of the grippers. The longer and wider arms of the XTW design is to enhance the capture and anchoring of the native leaflets between the arm and gripper (8). Studies have demonstrated that the MC G4 system outperforms its predecessors in reducing MR at 30 days, a reduction in the occurrence of device-associated clinical complications (12–15, 17).

**Figure 2:**
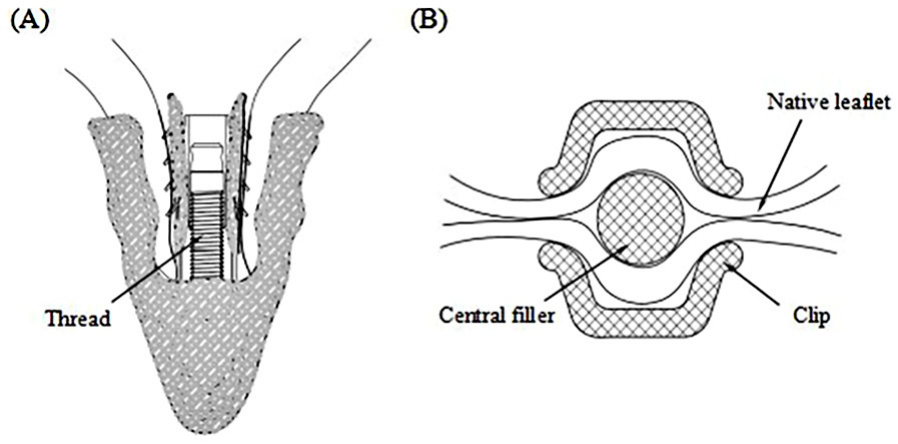
**(A)** GeminiOne self-locking design with threaded rigid central filler. **(B)** Native leaflets secured within the central filler and the wide “U-shaped” clip arms.

The Peijia Medical's GeminiOne (GEM) system (Peijia, Suzhou, China) is a novel TEER device; GEM0626 (long wide model) is shown in Figure 1B with a nominal arm length of 9 mm and a width of 6 mm and consists of four pairs of frictional element on each of the grippers. As illustrated in Figures 2A,B, GEM consists of a clip arm with a central rigid cylinder (filler) with a self-locking inner threaded design (Elgiloy alloy) and a pair of grippers (nitinol alloy). The inner threaded element, connected and manipulated by an external delivery control, drives the closing (or opening) of the clip arm. Once the leaflets are captured between the grippers and the U-shaped clip arms, the clip arms can be closed by manipulating the inner threaded element (increasing torque). The grasped leaflet will be increasingly compressed and secured between the clip arm and the rigid central filler.

The success of a TEER system in treating MR should be evaluated based on its acute reduction of MR, as well as its ability to achieve a complication-free sustainable MR reduction over the long term. In this study, porcine mitral apparatus was used to create DMR and FMR models, which were then used to evaluate the acute effectiveness of MR reduction by XTW and GEM0626 in a BDC pulsatile flow duplicator. Furthermore, detachment force measurements were performed on the anchored implants and porcine leaflets to compare the leaflet anchoring capabilities of the two TEER devices.

## 2 Materials and methods

### 2.1 Ethical statement

Ethics approval was not required for this study because this was an *ex vivo* study. Porcine hearts were sourced from a local abattoir in Suzhou county which are considered by-products of the food processing industry, thereby not requiring to undergo ethnic committee review by local law.

### 2.2 Experimental models

#### 2.2.1 Preparation for *ex vivo* model

Due to the similarity between the physiological structure of the porcine mitral valve and human mitral valve, porcine valves were selected for the *ex vivo* simulation experiments (18).

**Figure 3:**
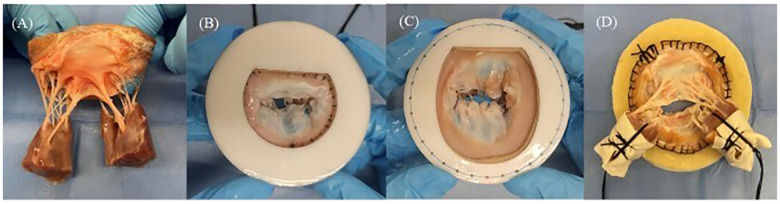
**(A)** Entire mitral valve apparatus excised from fresh porcine hearts. **(B)** Mitral annular matching felt layer and mitral disk for the degenerative mitral regurgitation model. **(C)** Mitral annular matching felt layer and mitral disk for the functional mitral regurgitation model. **(D)** Papillary muscle heads covered by fabric and wrapped by suture threads.

To prepare the MR model, porcine hearts were obtained, and the entire mitral valve apparatus, including both papillary muscle heads, all chordae tendineae, both leaflets, the annulus, and approximately 1 cm of left atrial tissue, was carefully excised (Figure 3A). The annular circumference was measured using a flexible ruler, and felt layers and 3D-printed mitral disks with inner circumferences close to the measured annular size were selected for the DMR model (Figure 3B). For the FMR model, felt layers and mitral disks with inner circumferences 50% larger than the annular size were selected (19), to create an enlarged mitral area with the primary extension between the anterior and posterior leaflets (Figure 3C). The annulus was sutured onto a felt layer glued to a mitral disk. The papillary muscle heads were wrapped with fabric material and polyester suture threads (Figure 3D).

#### 2.2.2 Preparation for leaflet captured area measurement and leaflet stability experiment

**Figure 4:**
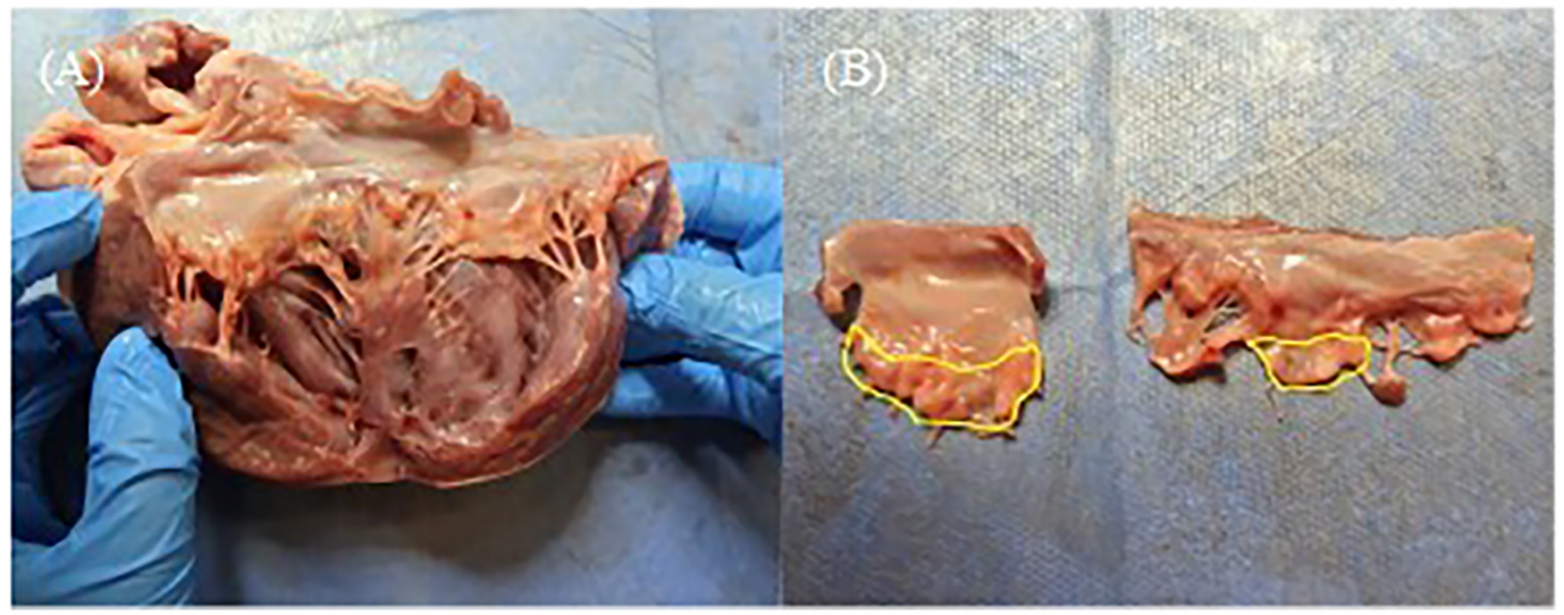
**(A)** The anterior and posterior leaflets were separated along the anterolateral commissures. **(B)** Complete anterior and posterior leaflets, and the yellow area is the rough zone.

The anterior and posterior leaflets were then separated along the anterolateral and posteromedial commissures (Figure 4A). As shown in Figure 4B, the anterior and posterior leaflets retained their complete rough and clear zone, as well as the annular portion (basal zone) (20). These leaflets were then used for the TEER device leaflet captured area experiment and leaflet anchoring stability experiment.

### 2.3 Pulsatile flow evaluation

**Figure 5:**
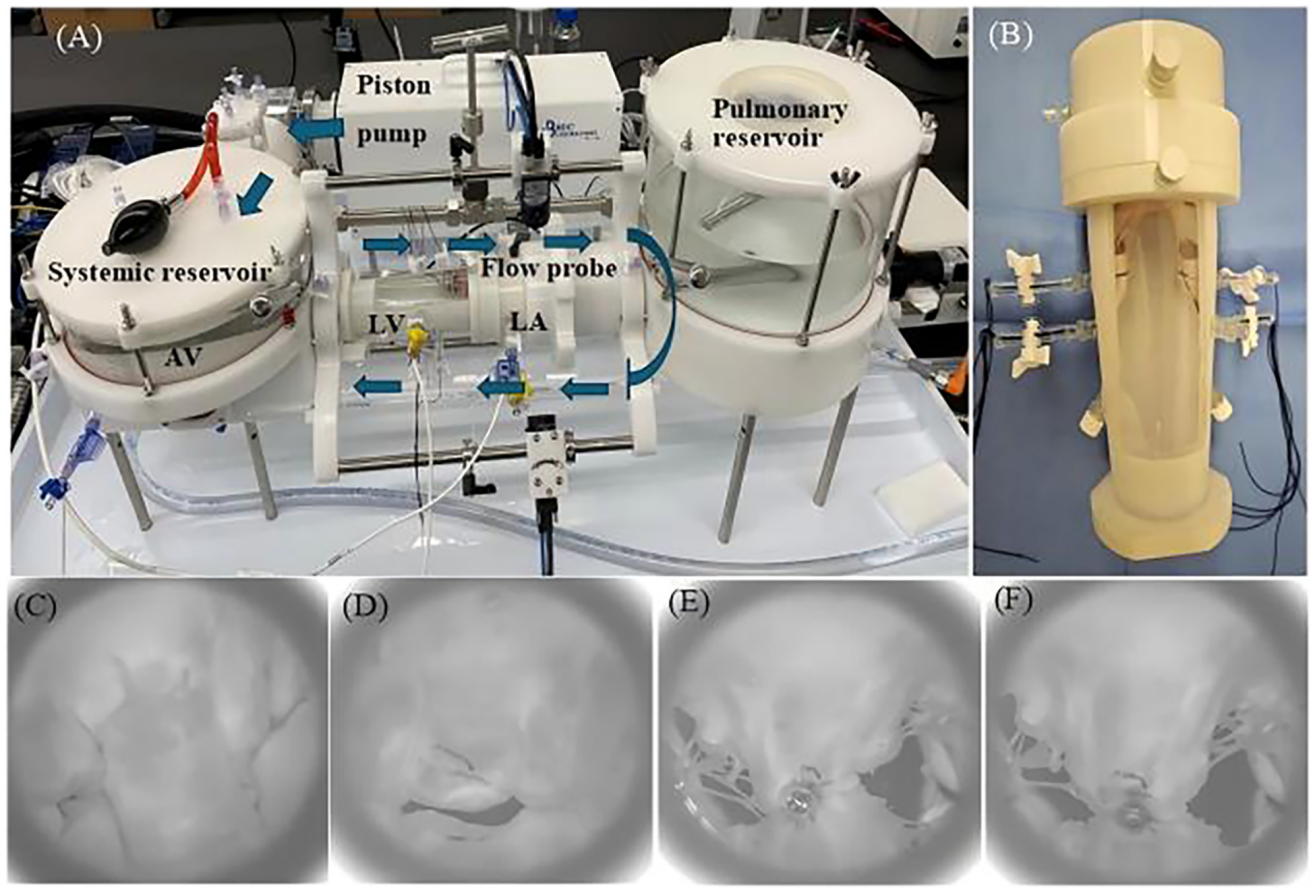
**(A)** Pulsatile flow duplicator device (arrows indicate the direction of flow). **(B)** The threads controlling the papillary muscles are fixed by two side channels on the left ventricle (LV) chamber. **(C)** *En face* view taken with high-speed after creating mitral valve flail of systolic. **(D)** *En face* view taken with high speed after creating functional mitral regurgitation. *En face* view after GeminiOne **(E)** and MitraClip **(F)** deployment [left atrium (LA), aortic valve (AV)].

The immediate efficacy of MR reduction by the two TEER devices was evaluated in an *ex vivo* mitral valve simulator (21, 22). This system (Figure 5A) has a piston pump that helps generate a physiological hydrodynamic environment for the mitral valve. An in-built transonic flow probe was used to monitor trans-mitral flow (ME 25PXNB, Transonic Systems Inc., Ithaca, NY, USA) and multiple pressure transducers (PT-7160, ifm electronic GmbH, Essen, North Rhine-Westphalia, Germany) were used to measurement the pressure inside the left ventricular, left atrial, and aortic chambers.

The prepared MR model was placed into the 3D-printed left ventricle (LV) chamber. Subsequently, the suture thread used to secure the papillary muscle was extruded through two side holes of the chamber and fixed first. Afterward, another 3D-printed left atrial chamber was attached to the left ventricle chamber along with the mitral valve (Figure 5B), and the entire setup was mounted onto the pulsatile flow duplicator (HDTi-6000 Heart Valve Pulse Duplicator, BDC Laboratories, Wheat Ridge, CO, USA).

The system was filled with 0.9% saline, and the hemodynamic setting for the mitral valve with 70 bpm, 5 L/min cardiac output, diastole occupying 2/3 of a cardiac cycle, and aortic pressure of 120/80 mmHg was set up. For the DMR, the papillary muscle heads were positioned anatomically below the commissures, and the marginal chordae tendineae of the P2 segment of the mitral valve's posterior leaflet were transected to establish the mitral valve flail model. The flail of the P2 segment was verified using a high-speed camera (Figure 5C), accompanied by recorded hydrodynamic data. For the FMR model, the suture threads attached to the papillary muscles were tightened to further restrict the mobility of the anterior and posterior leaflets. Combined with the expanded annulus, this created a gap at the coaptation zone between the anterior and posterior leaflets, as shown in Figure 5D. Hydrodynamic data were subsequently recorded in the same manner. Finally, a simplified short-handle TEER delivery catheter was used to deploy the TEER implants (GEM0626 and XTW) onto the both A2 and P2 segments (Figures 5E,F). The grasped positions on the leaflets were marked to ensure consistent positioning for both TEER devices and uniform lengths of the secured leaflets. The mitral valve was remounted into the duplicator and recorded the hydrodynamic data.

For the DMR and FMR, six sets of comparative experiments between XTW and GEM0626 were respectively conducted to evaluate the acute efficacy of MR reduction.

**Figure 8:**
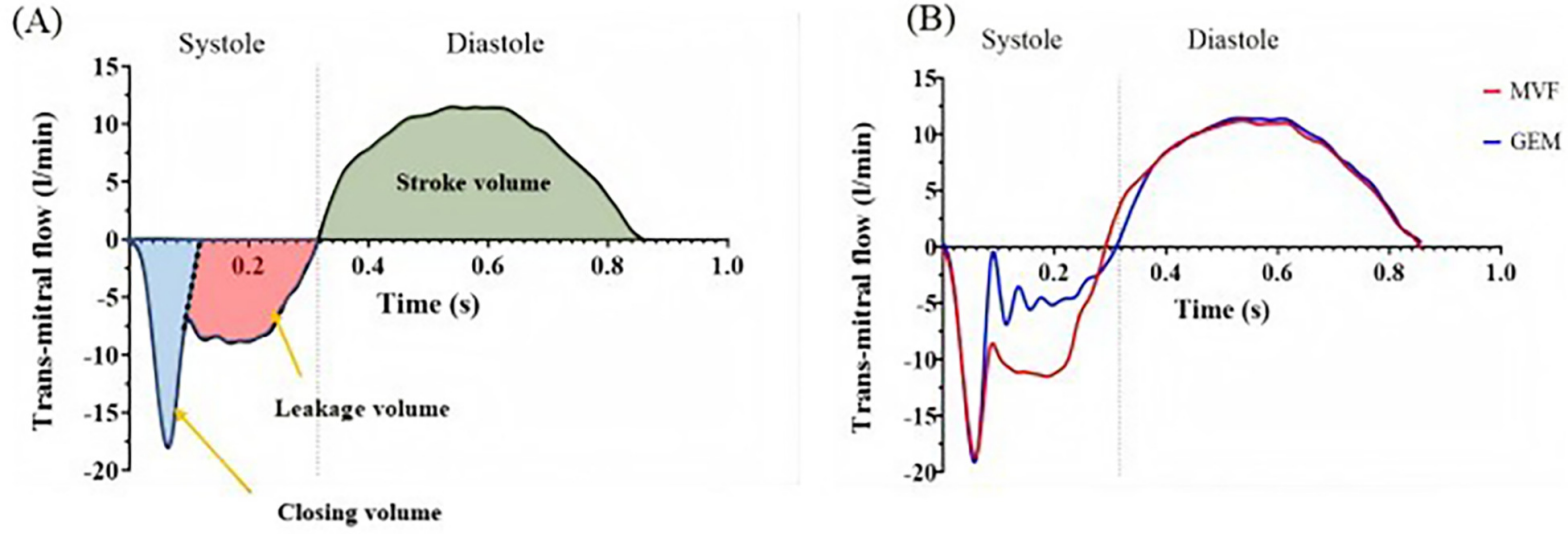
**(A)** Representative trans-mitral flow pattern. **(B)** Flow pattern of a single cardiac cycle acquired using a transonic flow probe under degenerative mitral regurgitation conditions [mitral valve flail (MVF), GeminiOne (GEM)].

After the simulator ran stably, all captured hemodynamic data were averaged over 10 cardiac cycles, and the trans-mitral flow and pressure data were analyzed. Figure 8A shows a representative trans-mitral flow waveform captured by the flow probe. During initial systolic valve closure, a certain volume of saline was pushed into the left atrial chamber, causing a negative flow pattern, which was calculated as the closing volume. The remaining negative flow during systole was integrated as the leakage volume. The positive flow during mitral valve opening in diastole was integrated as the forward flow, which is considered as the stroke volume. The regurgitant fraction was used to assess the severity of MR and was calculated as the ratio of the regurgitant volume (closing volume + leakage volume) to the total stroke volume, expressed as a percentage. The mean pressure difference (PD_mean_) between the left atrium and the left ventricle during diastole was recorded, and the effective orifice area (EOA) was calculated using the following equation (23):

$$\mathrm{EOA}=\frac{\mathrm{Qrms}}{51.6\sqrt{\frac{PD_{\mathrm{mean}}}{\rho }}}$$

where Qrms is the root mean square of diastolic forward flow and ρ is the density of the physiological saline.

### 2.4 Leaflet captured area comparison between TEER devices

**Figure 6:**
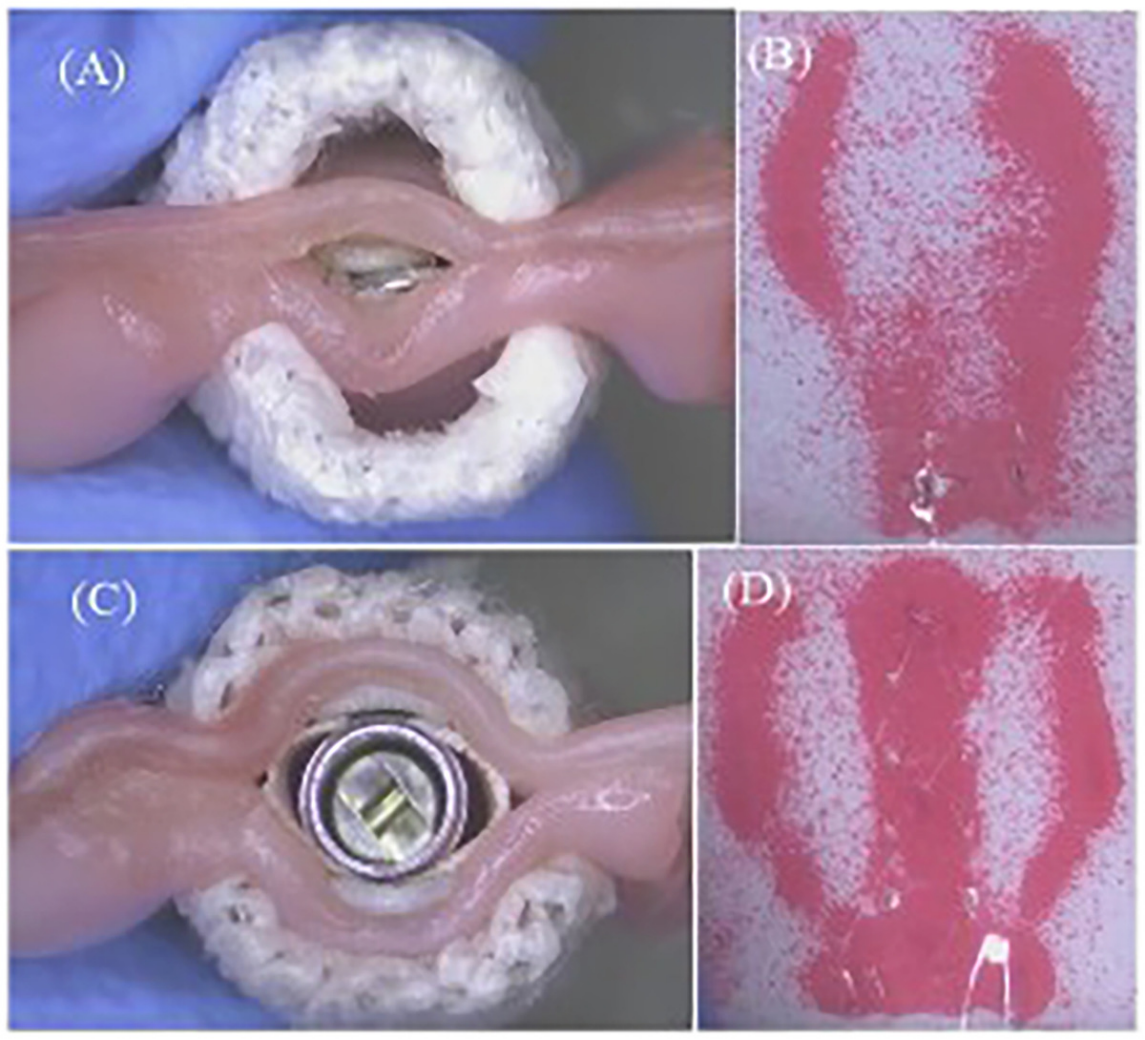
**(A)** Closed MitralClip XTW with anchored leaflets. **(B)** Corresponding pressure field (MitralClip). **(C)** Closed GeminiOne with anchored leaflets. **(D)** Corresponding pressure field (GeminiOne).

Due to the different designs of the clip arm structures of MC and GEM, the leaflet captured areas in contact with the clip arms at the closed configuration of the TEER device vary, as illustrated in Figures 6A,C. To further investigate whether the structural differences between the clips of the two TEER devices impact the degree of MR reduction, we first need to examine the leaflet area that the clip can accommodate.

Trimmed porcine leaflets were used as leaflet model in this experiment. The average thickness of the porcine mitral valve is 1.3 ± 0.3 mm.

XTW and GEM0626 were used to compare the captured area for different grasp lengths (*n* = 11). Area measurements were conducted for GEM0626 at a grasp length of 6 and 9 mm, while XTW measurements were taken at a grasp length of 6, 9 mm, and overall length (nominal 12 mm). Given that the pressure-sensitive films (0.5–2.5 MPa, FUJIFILM, Tokyo, Japan) exhibit a specific level of hardness and thickness, which may impact the contact area between the clip arms and the leaflets, the pressure-sensitive films are arranged between the grippers and the leaflets. When the clip is closed tightly, these films change color due to compression, indicating the captured area of the leaflet (Figures 6B,D). The outer frame of the discolored area was marked, and the contour of the unfolded leaflet was captured using an imaging measurement device, through which the contour area was determined by AutoCAD 2021 (Autodesk, Inc., San Francisco, CA, USA).

### 2.5 Leaflet anchoring stability of TEER devices

MR reduction relies on securing mitral leaflets within the “closed” TEER device, i.e., leaflet anchoring stability. The stability is influenced by the length of leaflets grasped by the clip arm, assessed by measuring the force needed to detach the secured leaflet under different grasp lengths.

Clinically, MC emphasizes the importance of capturing sufficient leaflet length (XTW nominal length of 12 mm recommended capturing at least 9 mm of leaflet length) (24). In this experiment, “partial grasp” is defined as a grasped leaflet length that does not meet the minimum capture length recommended. Under the partial grasp condition, due to the reduction in the contact between the clip and the leaflets, certain partial frictional elements on the gripper do not engage with the leaflets.

Since the TEER implant is clamped around a rough zone of the porcine valve leaflet (within the yellow area in Figure 4B), the surface of this zone connected to the chordae tendineae is very rough, resulting in a highly uneven thickness of this area. To control the experimental variables, in this study, the closing angle of the TEER implant for both fully and partially grasped leaflets was controlled to be approximately 12°.

#### 2.5.1 Experimental design

Hence, taking into account the abovementioned factors affecting the stability of the anchoring force, the following experiments are designed to explore the anchoring force stability of MC and GEM clips. Table 1 illustrates the experiment setup to compare the detachment force between XTW and GEM0626 (*n* = 9). For the “full grasp” comparison, the inserted leaflet length for both XTW and GEM0626 devices was in accordance with the respective nominal arm length. Whereas for the “partial grasp” comparison, XTW is set to 6 mm, which corresponds to 2/3 of its recommended grasping length (XTW is recommended to grasp at least 9 mm of leaflet length) (24). For GEM0626, “partial grasp” was also set to 6 mm, which corresponds to 2/3 of its nominal arm length.

**Table 1:** Experimental matrix for leaflet detachment force comparison between XTW and GEM0626 [GeminiOne (GEM)].

XTW (full grasp)	XTW (partial grasp)	GEM0626 (full grasp)	GEM0626 (partial grasp)
GLL	GLL	GLL	GLL
12 mm	6 mm	9 mm	6 mm

Experiments carried out under either fully grasped or partially grasped leaflets, defined with different grasping leaflet lengths (GLL). Sample size *n* = 9.

#### 2.5.2 Experimental setup

**Figure 7:**
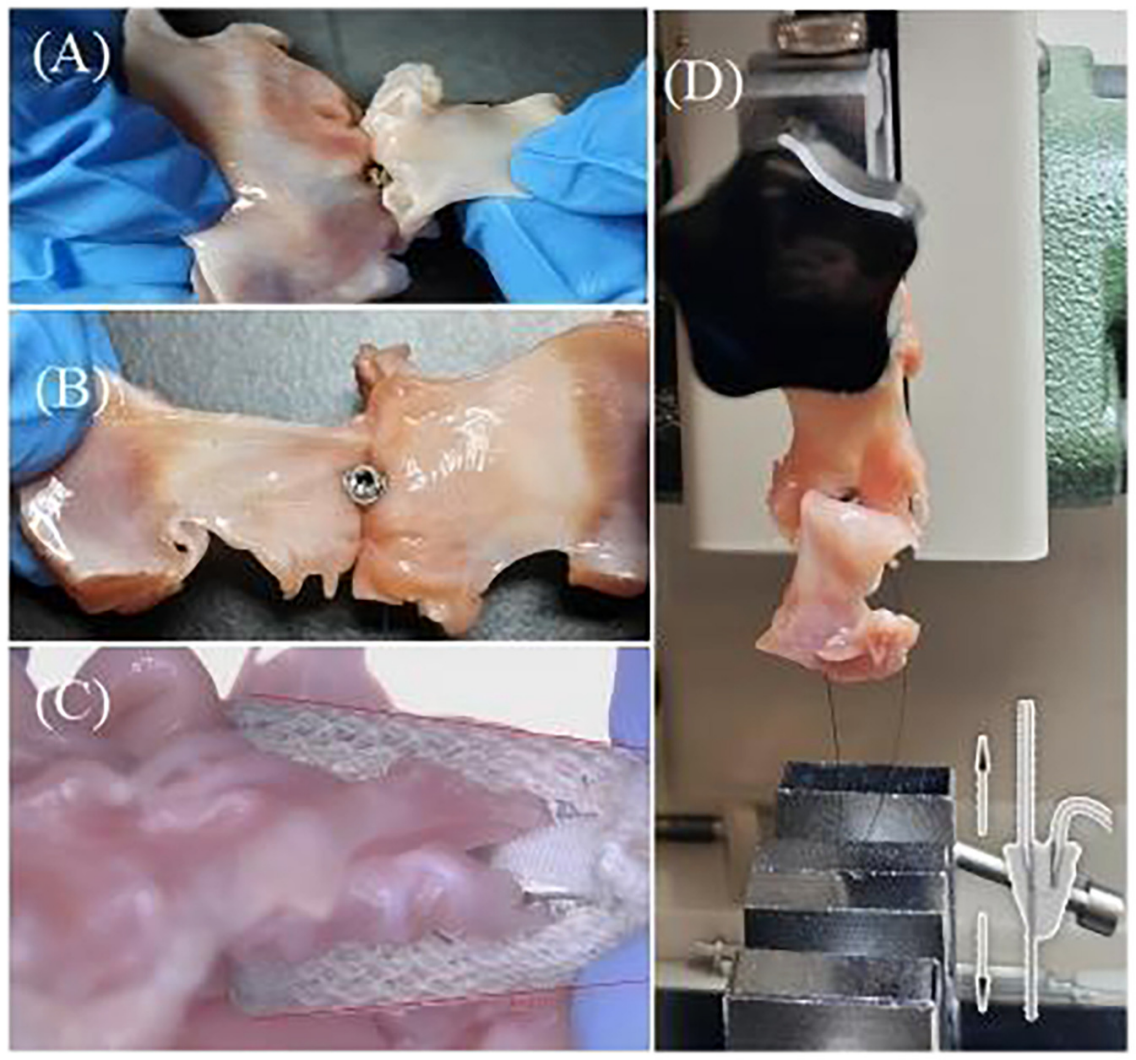
**(A)** Preparation of closed XTW samples for the detachment force experiment. **(B)** Preparation of closed GEM0626 samples for the detachment force experiment [GeminiOne (GEM)]. **(C)** Closed angle of GEM0626. **(D)** Experimental setup for detachment force measurements.

The setup procedure involves inserting the porcine mitral valve leaflet into both sides of the clip arms and then lowering the gripper to capture the leaflet. Subsequently, the clip will be manually closed and locked, with the fully closed state shown in Figures 7A,B. Optical imaging measurements (950HC, OUMIT Inc., Suzhou, China) were used to measure the angle between two clip arms of the TEER device with an anchored leaflet—closed angle, as shown in Figure 7C. The leaflet detachment force test is conducted by fixing the clip at one end of the tensile test fixture of the force gauge (HP-50, HANDPI Inc., Yueqing, China), while the leaflet is fixed on the other end (Figure 7D). The sample is pulled vertically along the length of the device, and the peak force at which the leaflet is detached from the device is recorded, defined as the detachment force.

### 2.6 Statistical analysis

Statistical analysis of this study is performed with GraphPad Prism 9.0 (GraphPad Software, San Diego, CA, USA). For *ex vivo* hemodynamic evaluation, the data were first tested for normality. Data that follow normal distribution were presented as mean ± standard deviation and analyzed by paired *t* test. Data that failed to pass the normality test were presented as median with interquartile ranges and analyzed by the Wilcoxon test. A *p*-value of ≤0.05 is considered as statistically significant.

## 3 Results

### 3.1 Pulsatile flow evaluation

Figure 8B shows the measured flow pattern of a single cardiac cycle acquired using a transonic flow probe under DMR conditions. This pattern is consistent with the typical flow patterns illustrated in Figure 8A. Therefore, the *ex vivo* mitral valve simulator can be used to quantify regurgitation under different pathological conditions.

#### 3.1.1 DMR model

**Figure 9:**
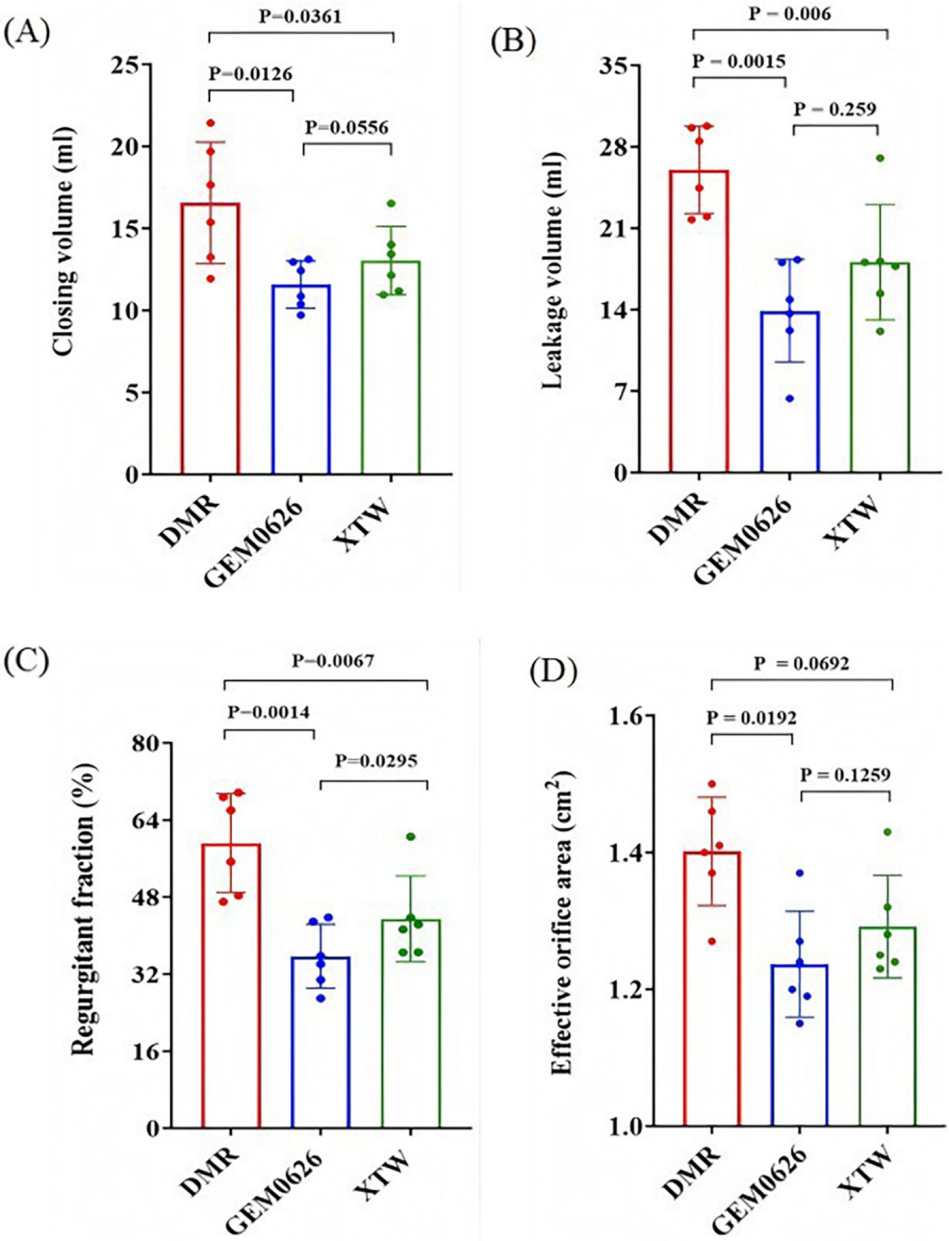
Pulsatile flow evaluation between GeminiOne (GEM) and MitraClip under degenerative mitral regurgitation (DMR) experimental conditions. Sample size of *n* = 6. **(A)** Closing volume. **(B)** Leakage volume. **(C)** Regurgitant fraction. **(D)** Effective orifice area.

As shown in Figures 9A,B, significant reductions in both closing volume and leakage volume were observed in the presence of GEM and MC as compared with the DMR condition. Specifically, the closing volume: GEM0626: 16.55 ± 3.70 vs. 11.57 ± 1.45 ml, *p* = 0.0126; XTW: 16.55 ± 3.70 vs. 13.04 ± 2.09 ml, *p* = 0.0361. The leakage volume: GEM0626: 26.01 ± 3.75 vs. 13.91 ± 4.41 ml, *p* = 0.0015; XTW: 26.01 ± 3.75 vs. 18.08 ± 4.96 ml, *p* = 0.006. The overall regurgitant fraction (Figure 9C) further demonstrates the trend (GEM0626: 59.21 ± 10.29% vs. 35.73 ± 6.62%, *p* = 0.0014; XTW: 59.21 ± 10.29% vs. 43.50 ± 8.89%, *p* = 0.0067). GEM seems to have a slightly better capability of lowering regurgitant fraction (GEM0626 vs. XTW: 35.73 ± 6.62% vs. 43.50 ± 8.89%, *p* = 0.0295).

The impact of XTW and GEM0626 TEER devices on mitral valve diastolic hemodynamics is illustrated in Figure 9D. The presence of both GEM0626 and XTW lower the EOA compared with DMR (GEM0626: 1.40 ± 0.08 vs. 1.24 ± 0.08 cm^2^, *p* = 0.0192; XTW: 1.40 ± 0.08 cm^2^ vs. 1.29 ± 0.08 cm^2^, *p* = 0.0692).

#### 3.1.2 FMR model

**Figure 10:**
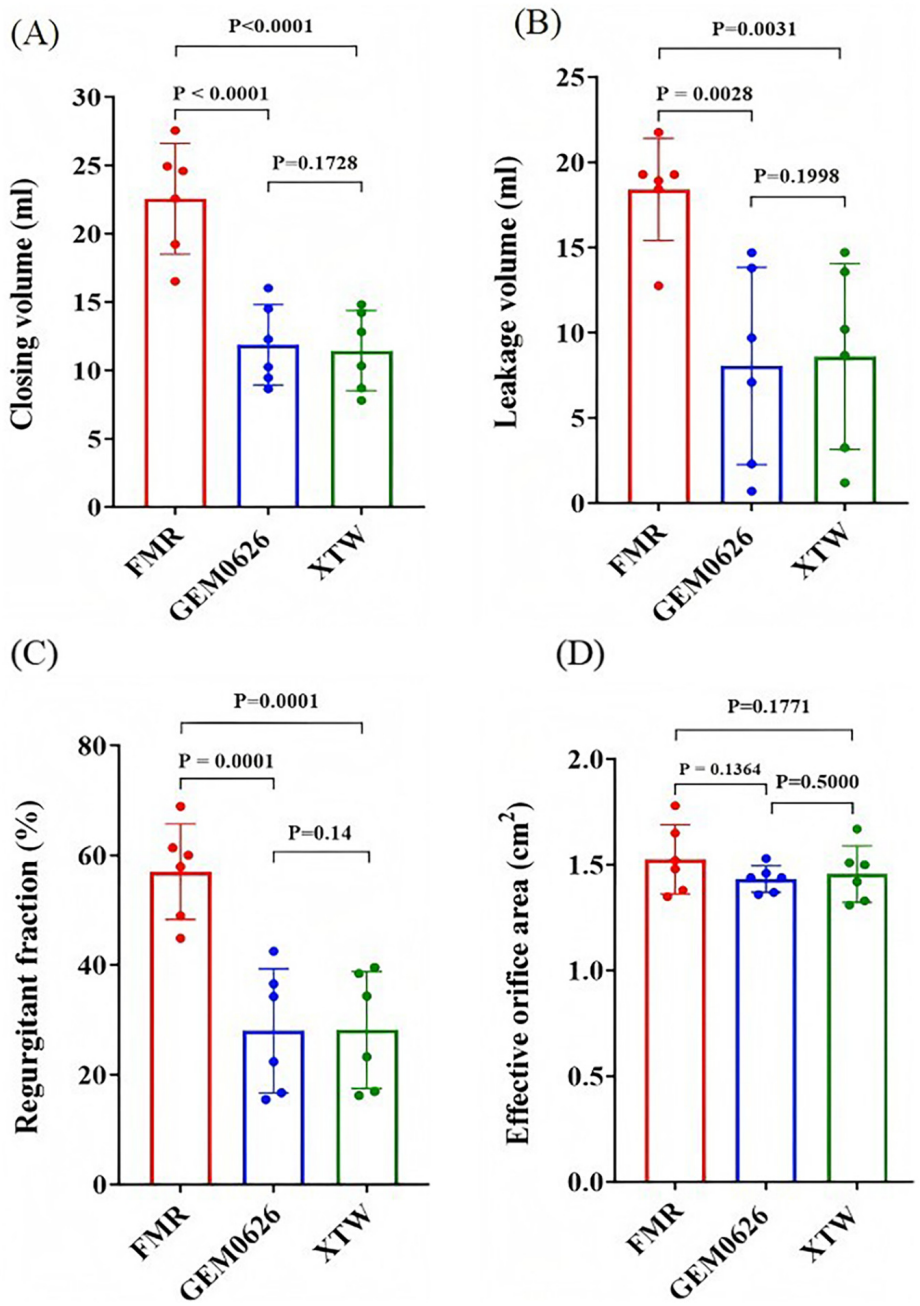
Pulsatile flow evaluation between GeminiOne (GEM) and MitraClip under functional mitral regurgitation (FMR) experimental conditions. Sample size of *n* = 6. **(A)** Closing volume. **(B)** Leakage volume. **(C)** Regurgitant fraction. **(D)** Effective orifice area.

The pulsatile flow evaluation for the FMR model is illustrated in Figure 10. The results indicate that both GEM0626 and XTW significantly reduced closing volume, leakage volume, and regurgitant fraction. The closing volume (Figure 10A): GEM0626: 22.56 ± 4.06 vs. 11.87 ± 2.94 ml, *p* < 0.0001; XTW: 22.56 ± 4.06 vs. 11.45 ± 2.93 ml, *p* < 0.0001. The leakage volume (Figure 10B): GEM0626: 18.40 ± 3.00 vs. 8.05 ± 5.80 ml, *p* = 0.0028; XTW: 18.40 ± 3.0 vs. 8.60 ± 5.44 ml, *p* = 0.0031. The regurgitant fraction (Figure 10C): GEM0626: 56.99 ± 8.74% vs. 27.99 ± 11.30%, *p* = 0.0001; XTW: 56.99 ± 8.74% vs. 28.13 ± 10.64%, *p* = 0.0001. For the comparison of EOA (Figure 10D), no significant differences were observed among FMR, GEM0626, and XTW (1.53 ± 0.16 vs. 1.43 ± 0.06 vs. 1.46 ± 0.13 cm^2^).

Comparing the results of DMR and FMR, the effect of the TEER device led to a more significant reduction in closing volume for FMR compared with DMR, while the degree of leakage volume reduction was similar for both.

### 3.2 Leaflet captured area comparison between TEER devices

**Figure 11:**
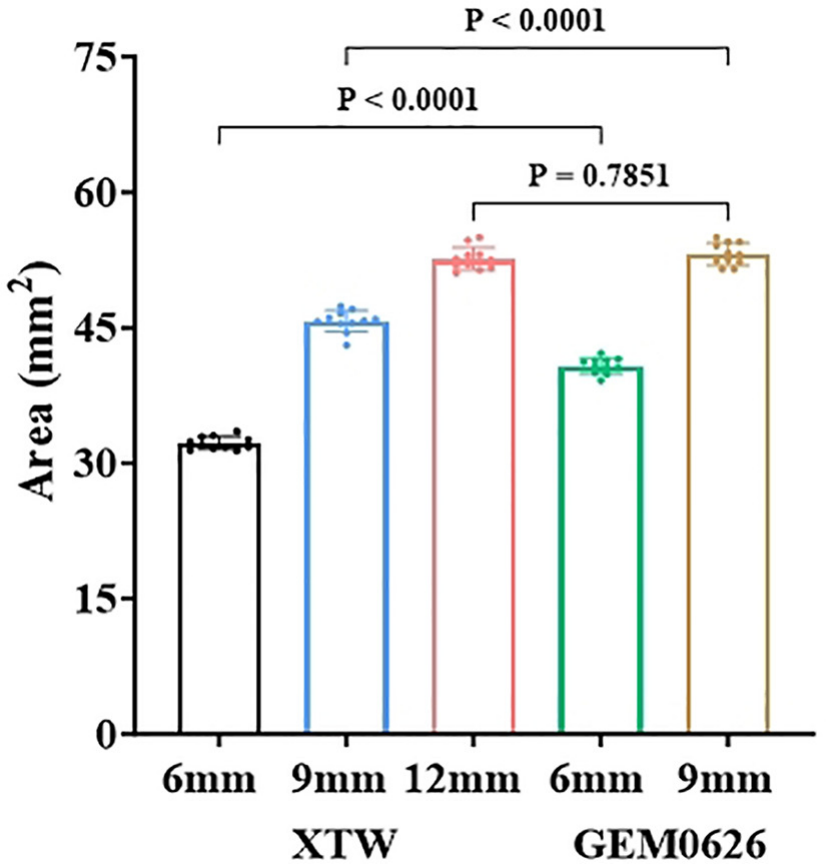
The leaflet captured area of the GeminiOne (GEM) and MitraClip were measured and compared under full grasp and partial grasp conditions. Sample size of *n* = 11 (mean ± SD).

The comparison of the different leaflet captured areas between the two TEER devices was illustrated in Figure 11. It can be seen that “GEM0626 9 mm” has the largest captured area, compared with “XTW 12 mm” which has a slightly smaller captured area, with no significant difference (53.15 ± 1.23 vs. 52.62 ± 1.26 mm^2^, *p* = 0.7851). In contrast, the captured area of “XTW 9 mm” is comparatively smaller than “GEM0626 9 mm” (45.70 ± 1.20 vs. 53.15 ± 1.23 mm^2^, *p* < 0.0001). On the other hand, comparing the partial grasp condition, the captured area of “XTW 6 mm” is significantly smaller than “GEM0626 6 mm” (32.22 ± 0.72 vs. 40.77 ± 0.86 mm^2^, *p* < 0.0001). The above results indicate that, with the same length of grasped leaflet, GEM0626 secured more leaflet tissue compared with XTW.

### 3.3 Leaflet anchoring stability evaluation

**Figure 12:**
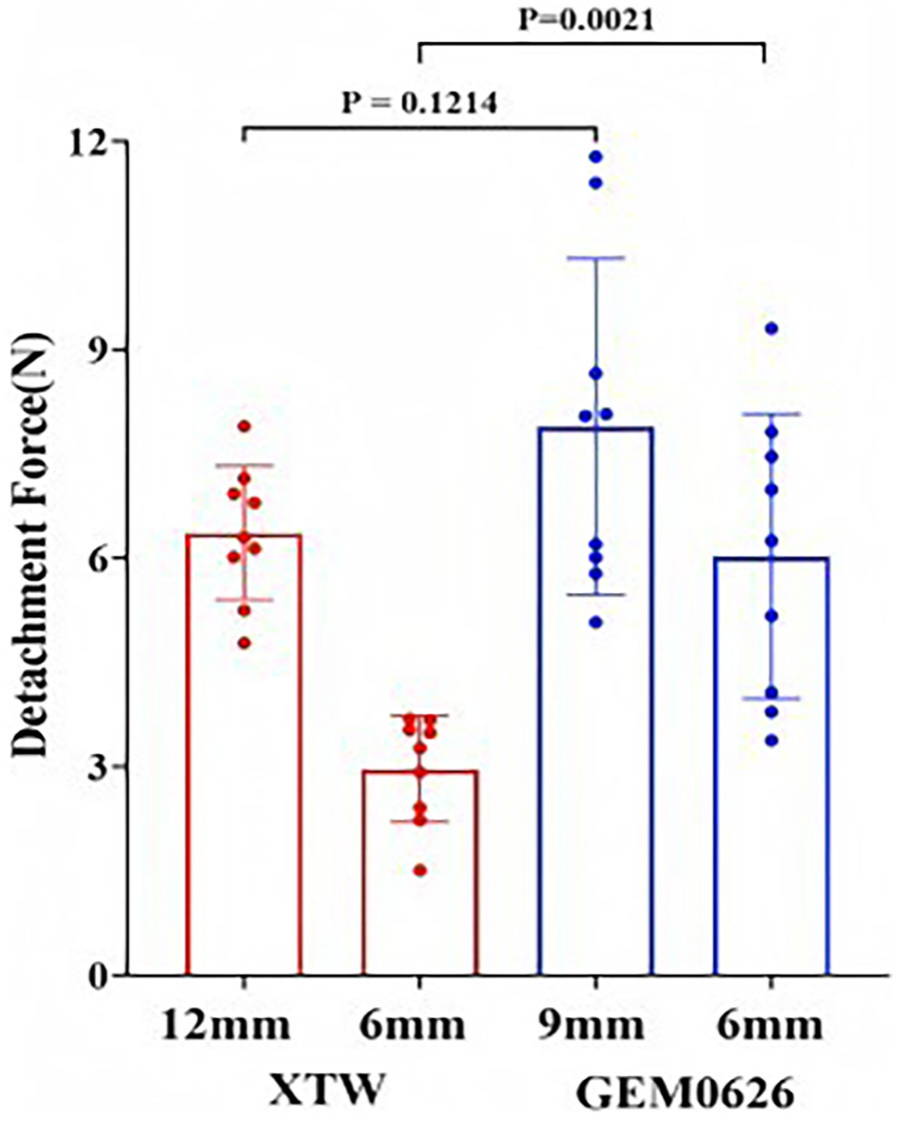
Transcatheter edge-to-edge repair anchored leaflet stability evaluation. Detachment force was evaluated for the GeminiOne (GEM) and MitraClip under full grasp and partial grasp conditions. Sample size of *n* = 9 (mean ± SD).

Detachment force evaluation between XTW and GEM0626 under full grasp and partial grasp conditions were shown in Figure 12. Comparing the results of full and partial grasp for XTW, the average detachment force decreased significantly from 6.36 to 2.97 N. The trend shows that having a shorter inserted leaflet length results in a lower detachment force of leaflets in XTW. However, GEM0626 demonstrated a different pattern compared with XTW, showing only a slight decrease in results when transitioning from full grasp to partial grasp (7.89 vs. 6.03 N). In addition, Figure 12 illustrates the comparison of detachment force between GEM0626 and XTW (full grasp detachment force: 7.89 ± 2.42 vs. 6.36 ± 0.96 N, *p* = 0.1214; partial grasp detachment force: 6.03 ± 2.05 vs. 2.97 ± 0.76 N, *p* = 0.0021). Under the partial grasp condition, the detachment force of GEM0626 is more than twice that of XTW. Under the full grasp condition, the detachment force of GEM0626 is slightly higher than that of the MitraClip but without a statistically significant difference.

## 4 Discussion

In this project, using *ex vivo* benchtop setups, we evaluated MR correction efficacy and mitral leaflet anchoring stability between two TEER devices, namely, MitraClip and GeminiOne. The key findings can be summarized as follows: First, GEM has better regurgitation correction capabilities than MC in the DMR model, but not in the FMR model. Second, GEM has a larger leaflet-capturing area and higher leaflet detachment force compared with MC, suggesting improved tissue anchoring stability.

TEER with MC devices has been widely established as a viable treatment for MR patients with high surgical risk. However, adverse device complications due to unstable leaflet capturing such as SLDA and LLI are reported to occur in 1.6%–4.8% of the cases, which leads to the recurrence of MR that significantly impair patient outcomes (9–12). In addition, patients with wide areas of mitral leaflet lesions often have large regurgitant orifice areas that necessitate the implantation of more than one MC device (8), which is reported to impair diastolic hemodynamics. Building upon the unmet clinical need, we have developed a novel TEER device that can grasp a large leaflet area with stronger anchoring forces, lowering the likelihood of SLDA and LLI and the chances of needing a second device with comparable or improved MR correction compared with MC.

*Ex vivo* pulsatile models using porcine or ovine mitral valves have been widely reported in literature. Various leaflet geometric distortions such as prolapsed leaflet, annular dilation, and papillary muscle displacement that are seen clinically can be introduced in such models, which then allows for the investigation of any surgical procedures and transcatheter devices (21, 22). In our experiments, we successfully established clinically severe levels of DMR and FMR. Interestingly, despite similar regurgitant fraction results (DMR vs. FMR: 59.21 ± 10.29% vs. 56.99 ± 8.74%), the closing volume in the FMR model was larger than in the DMR model (16.55 ± 3.7 vs. 22.56 ± 4.06 ml), while the leakage volume was smaller in the FMR model (26.01 ± 3.75 vs. 18.40 ± 3.0 ml). This is likely due to delayed coaptation due to leaflet tethering in FMR. Such delay is reported to increase the backflow during the valve closure period (i.e., closing volume) (25).

Additionally, comparing the DMR models between the two TEER devices (Figure 9), GEM0626 was slightly better than XTW in terms of mean regurgitation reduction. Based on the leaflet captured area experiments, this phenomenon might be explained by the fact that, with the same length of leaflet grasped, GEM0626 captured more leaflet tissue than XTW, thereby eliminating a larger flail area. Similarly, since FMR does not involve prolapse or other leaflet abnormalities, the results for the two devices were very similar. However, in both DMR and FMR cases, both devices leave quite significant residual regurgitation, which is likely due to the overly severe regurgitation states created. Future studies will focus on comparing multiple devices.

The results of the leaflet captured area experiment also supported the observation in Figures 6A,B: The U-shaped cross-section of GEM0626's clip is wider and deeper compared with XTW, making it more capable of accommodating and compressing more leaflet tissue.

From the results of the leaflet anchoring stability experiment (Figure 12), both TEER implants were able to effectively anchor onto the leaflets when sufficient leaflet tissue was grasped. However, when only a partial grasp was achieved, the anchoring stability of XTW significantly decreased, while GEM0626 exhibited only a slight decline. This difference might be due to XTW relying solely on the frictional element on the gripper to stabilize the leaflet. Under partial grasp conditions, the shortened leaflets are engaged with lesser frictional elements, therefore resulting in a significantly lower engagement force compared with full grasp. On the other hand, GEM relies on both the frictional element on the gripper and the compression action between the clip arms and the rigid central filler. During clip closure, as the threaded structure inside the central filler is continuously tightened, the compression on the leaflet between the clip and the central filler gradually increases, providing additional stabilization (even with shorter grasp lengths) and preventing the leaflet from slipping out of the clip. This “screw thread” principle is similar to the screw jack; according to the principle of “The Mechanics of Power Screws” (26), torque (*T*) is linearly proportional to the compressive force (*F*), as illustrated by the equation as follows:

$$T=\frac{d_{m}}{2}\left(\frac{l+\pi fd_{m}}{\pi d_{m}-fl}\right)F,\;T=kF$$

where “l” is the lead of the thread, $d_{m}$ is the screw diameter, and *f* is the coefficient of friction. These parameters are determined by the material and size of the screw which can be simplified as constant *k*.

As discussed in the previous section, residual MR and certain complications (such as SLDA and LLI) are common causes of MR recurrence. This study evaluated the pulsatile flow and leaflet anchoring of two TEER implants, offering insights for clinical applications. When using XTW and GEM0626 to treat DMR, the clip should aim to grasp as much prolapsed leaflet tissue as possible to further reduce systolic leakage volume. In addressing FMR, efforts should focus on coapting the anterior and posterior leaflets at the implant location to counteract the tethering effects from the papillary muscle and annular dilation, ultimately eliminating the delayed coaptation of leaflets. By achieving a good leaflet coaptation, residual regurgitation can be minimized, enabling patients to achieve long-term clinical benefits. Furthermore, in cases of either incomplete or inadequate leaflet grasping, TEER implants with robust leaflet anchoring stability can reduce the risk of SLDA and LLI, which therefore leads to design features that enhance such property, for example, by incorporating more frictional elements on the gripper or optimizing their distribution. Alternatively, additional stabilization could be provided, as seen with GEM, through leaflet compression.

Additionally, comparing GEM0626 and XTW for FMR models, there is significantly higher leaflets tension when the TEER is coapted with the leaflets as compared with DMR models due to the much higher opposing forces from the annular dilation and chordal tethering. In some of the FMR model experiments, full leaflet grasping resulted in excessive tension which led to incomplete clip closure. This finding may be used to explain why clinical reports indicate that MitraClip G4 NTW (with an arm length of 9 mm and four pairs of frictional elements on the gripper) is the most frequently used for FMR (17). Using shorter arm lengths frees up more leaflet tissue, hence reducing the tension on the leaflets. On the other hand, GEM, with its better anchoring stability at shorter grasp lengths, leads to reduced tension on the mitral leaflets and potentially lowers the risk of TEER-induced leaflet injury. Further clinical validation is required to confirm these findings.

## 5 Study limitations

There are a few limitations in this research. First, the *ex vivo* mitral valve study is carried out by simulating the hemodynamics of the mitral valve without considering the dynamic motion of the mitral annulus, the viscosity of blood, papillary muscle, and actively contractile components of the left atrium and left ventricle. Second, the valvular diseased model is either DMR (P2 segment) or FMR; the efficacy of regurgitation correction by GEM has not been studied in other MR etiologies such as myxomatous degeneration and commissural flail. Third, the FMR model is created by suturing the mitral apparatus onto an enlarged rigid 3D-printed disk which does not allow any annular remodeling after TEER implantation which differs from the clinical situation in some cases. Finally, the other commercial TEER device—Pascal (Edwards Lifesciences Corporation, Irvine, CA, USA) TEER was not compared in this analysis as the device is not available commercially in this jurisdiction.

## 6 Conclusion

In the *ex vivo* pulsatile experiments, both GEM0626 and XTW are effective in terms of acute reduction of MR caused by DMR and FMR. However, in an anchored leaflet stability study, especially under partial grasp conditions, GEM0626 demonstrated significantly higher leaflet detachment force. The better anchored stability of GEM TEER may have long-term clinical benefits for MR treatment.

## Data Availability

The raw data supporting the conclusions of this article will be made available by the authors, without undue reservation.
